# Root cause analysis and strategies for reducing falls among inpatients in healthcare facilities: A narrative review

**DOI:** 10.1002/hsr2.2216

**Published:** 2024-06-27

**Authors:** Parvin Lakbala, Najmeh Bordbar, Yadolah Fakhri

**Affiliations:** ^1^ Department of Health Services Management Hormozgan University of Medical Sciences Bandar Abbas Iran; ^2^ Health Human Resources Research Centre, School of Management and Medical Information Sciences Shiraz University of Medical Sciences Shiraz Iran; ^3^ Department of Environmental Health Engineering Hormozgan University of Medical Sciences Bandar Abbas Iran

**Keywords:** accidental falls, accident prevention, hospitalization, patient safety, Root Cause Analysis

## Abstract

**Background and Aims:**

Root Cause Analysis (RCA) is a systematic process which can be applied to analyze fall incidences in reactive manner to identify contributing factors and propose actions for preventing future falls. To better understand cause of falls and effective interventions for their reduction we conducted a narrative review of RCA and Strategies for Reducing Falls among Inpatients in Healthcare Facilities.

**Methods:**

In this narrative review, databases including Scopus, ISI Web of Science, Cochrane, and PubMed were searched to obtain the related literature published. Databases were searched from January 2005 until the end of March 2023. The Joanna Briggs Institute (JBI) tool was used for quality assessment of articles. To analyze the data, a five‐stage framework analysis method was utilized.

**Results:**

Seven articles that fulfilled the inclusion criteria were identified for this study. All of the selected studies were interventional in nature and employed the RCA method to ascertain the underlying causes of inpatient falls. The root causes discovered for falls involved patient‐related factors (37.5%), environmental factors (25%), organizational and process factors (19.6%), staff and communication factors (17.9%). Strategies to reduce falls involved environmental measures and physical protection (29.4%), identifying, and displaying the causes of risk (23.5%), education and culturalization (21.6%), standard fall risk assessment tool (13.7%), and supervision and monitoring (11.8%).

**Conclusion:**

the findings identify the root causes of falls in inpatient units and provide guidance for successful action plan execution. Additionally, it emphasizes the importance of considering the unique characteristics of healthcare organizations and adapting interventions accordingly for effectiveness in different settings.

## 1 INTRODUCTION

Falls are a primary cause of avoidable injury in hospital and outpatient settings,^1^ and can result in reduced mobility, functionality, and engagement in everyday tasks. Additionally, falls can contribute to longer hospital stays^2^, ^3^, ^4^ and increased mortality rates.^2^, ^3^, ^5^, ^6^ These incidents primarily affect frail patients, many of whom have dementia.^7^ Research indicates that the occurrence of falls can range from 1.7 to 16.9 falls per 1,00 patient days.^8^, ^9^ Among patients over the age of 65, approximately one‐third experience at least one fall per year.^10^ Furthermore, 20%–30% of falls result in moderate to severe injuries,^11^ such as skin excoriation, fractures, dislocations, and head trauma. Various factors contribute to the risk of falls, including age, gender, comorbidities, physical and psychosocial impairments, and medication usage. These risk factors are applicable to both the general population and the elderly.^12^, ^13^ Falls indeed create a substantial financial burden on the U.S. health systems, with an estimated $754 million in 2015 spent on medical expenses associated with fall‐related fatalities.^14^ Furthermore, approximately 43% of these incidents result in harm to the patient.^15^, ^16^ Approximately 14% of falls that occur in hospitals can be categorized as accidental due to environmental factors, such as slipping on a wet floor. Another 8% of falls are considered unpredictable, resulting from sudden disturbances in the patient's physical condition that affect their balance. The majority, accounting for 78% of falls, are deemed predictable.^17^ Potential factors that may contribute to falls include the introduction of new medications and alterations in medication dosage.^18^

Root cause analysis (RCA) was introduced in the healthcare industry nearly two decades ago. It is a methodical and structured approach employed to ascertain the fundamental factors that contribute to variations in performance. RCA serves as a widely accepted and standardized technique for pinpointing the causes of medical errors, thereby empowering healthcare establishments to devise strategies aimed at mitigating future errors.^19^ RCA has been widely employed within healthcare settings, particularly in hospitals, to systematically address the issue of falls.^20^ The primary objective of RCA is to identify and rectify the underlying cause of falls, thereby mitigating the likelihood of recurrence.^21^ It is important to note that the primary objective of the RCA process is not to assign blame to individuals, but rather to identify shortcomings in systemic processes, with the ultimate goal of preventing harm to patients, minimizing the occurrence of adverse events, and rectifying deficiencies.^22^

Studies have shown that implementing a fall prevention program based on RCA can lead to positive outcomes for inpatients and enhance patient safety through subsequent interventions.^23^, ^24^ While single and multi‐component interventions are planned to reduce hospital falls, data on successful prevention programs are limited.^20^ On the other hand, narrative reviews play a crucial role in presenting a descriptive summary of a topic and conducting a subjective analysis of the literature. They are essential for synthesizing complex research evidence in a detailed and nuanced manner, particularly for topics requiring comprehensive analysis. Narrative reviews offer a flexible and practical approach to synthesizing diverse literature, making them valuable tools.^25^, ^26^ Therefore, the aim of this narrative review study was to identify the causes of falls and strategies for their reduction within inpatient healthcare settings through RCA.

## 2 METHODOLOGY

### 2.1 Search strategy

In this narrative review, databases including Scopus, ISI Web of Science, Cochrane, and PubMed were searched to obtain the related literature published. Databases were searched from January 2005 until the end of March 2023. The search strategy included the following keywords: ‘RCA,’ ‘Root Cause Analysis,’ Falls, ‘Patient falls,’ ‘Reduce Falls,’ ‘Preventable Falls,’ ‘Decreasing Falls,’ ‘Patient safety,’ Safety, Patient, Delivery, Problems.” In order to increase sensitivity (to increase the selection of related articles), the researcher searched several databases with relatively common terms and synonymous words using the “OR” operator in the titles and abstracts of articles. In addition, to increase the specificity (to reduce the selection of irrelevant articles), we used “AND” operator. The search strategy is given in Table 1. In addition, to ensure a comprehensive search of articles, the references of selected articles were also reviewed.

**Table 1:** The search strategy.

**Search Engines and Databases:** Scopus, ISI Web of Science, Cochrane, and PubMed	
**Limits:** Language (English) and only original research	
**Date:** January 2005 ‐ March 2023	
**Strategy**: #1 AND #2, #1 AND #3	
#1	“RCA” OR “root cause analysis”
#2	“Patient safety” OR “delivery” OR “safety” OR “patient” OR “Problems”
#3	“falls” OR “reduce* falls” OR “decreasing falls” OR “patient falls” OR “preventable falls”

### 2.2 Inclusion and exclusion criteria

The following criteria were used as guidelines for screening articles. The inclusion criteria for this study focused on selecting original English articles that employed RCA methods and were interventional in nature. Articles from Journals that did not have a rigorous evaluation process, book reviews, opinion articles, review articles, and letters to editor were exclusion criteria.

### 2.3 Study selection

The studies obtained from the search were screened by two reviewers (P.L and N.B) separately in three stages (title, abstract and full text), and the result was based on the agreement of these two reviewers. Third reviewer, Y.F., helped to reach a consensus regarding the disagreement. Screening was performed using Endnote v.8.

### 2.4 Critical appraisal

The Joanna Briggs Institute (JBI) tool was used for quality assessment of articles. We used the JBI Statement, which has a 9‐item checklist, to ensure transparent reporting of studies. Each study was assessed by indicating “Yes,” “No,” “Unclear” or “Not applicable” for each item on the checklist.^27^ To prevent bias, the assessments were performed by two reviewers (Parvin Lakbala and Najmeh Bordbar), and a consensus was reached through Third reviewer, Yadolah Fakhri.

### 2.5 Data extraction

According to the screened studies, the data were extracted to achieve the research goals and questions. For this purpose, first the data extraction form including the names of authors of the article, year, country of study, health setting, health units, type of study, study design, RCA participants, intervention period, fall reduction, fall causes and reducing strategies was designed. In the next step, one of the authors (Parvin Lakbala) extracted the data from the selected articles and the second author (Najmeh Bordbar) reviewed the data. This form was designed and completed for each article in Excel 2013.

### 2.6 Data synthesis

To analyze the data, a five‐stage framework analysis method was utilized. In the first stage, the researcher repeatedly read extracted data from the included articles to become familiar with the data. The second stage involved forming repetitive ideas into groups of similar themes to identify a thematic framework. The third stage, indexing, focused on identifying units or clusters of data related to specific themes. Following indexing, the data were summarized in a table of themes based on the thematic framework. In the fifth stage, the data were combined, and maps and interpretations were used to clarify concepts, illustrate relationships between concepts, characterize the phenomenon, and provide explanations and suggestions.^28^ To ensure dependability, two members of the research team (Parvin Lakbala and Najmeh Bordbar) individually analyzed the contents and discussed the issues to reach a consensus. Ultimately, at the end of this process, the causes of falls and strategies for their reduction were identified.

## 3 RESULTS

**Figure 1:**
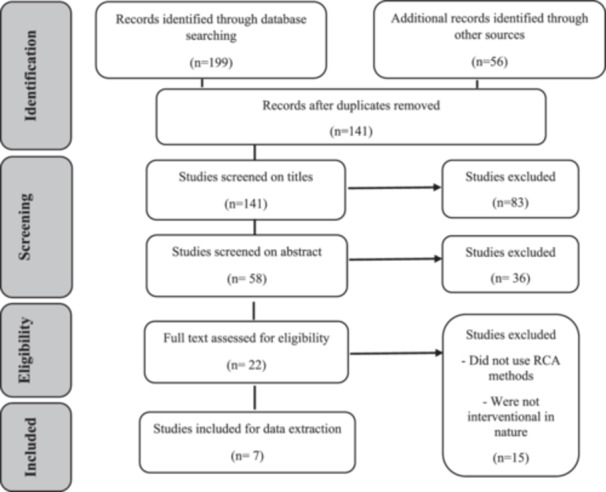
PRISMA Flow diagram for article selection.

In the initial search, 255 articles were found and after removing duplicate and irrelevant articles, the abstract of 58 articles was reviewed. At this stage, 36 articles were deleted since they did not meet the inclusion criteria. Finally, after reading the full text of 22 articles, only seven articles were eligible for inclusion (Figure 1).

Table 2 presents the characteristics of the seven included studies. Studies were conducted in the United States^6^ and Saudi Arabia,^1^ published between 2008 and 2022. Four studies were conducted in a general hospital (inpatient units),^29^, ^31^, ^33^, ^34^ 1 study in a children's hospital (mother and baby unit),^30^ 1 study in an acute care hospital (inpatient behavioral unit),^23^ and 1 study in a level one trauma center (emergency department).^32^ All studies were interventional and used the RCA method to identify the causes of inpatients' falls. ^23^, ^29^, ^30^, ^31^, ^32^, ^33^, ^34^ Most studies had established interdisciplinary quality improvement teams and committees to identify causes of falls,^23^, ^29^, ^30^, ^31^, ^33^, ^34^ and only one study had revised an existing risk assessment tool.^32^ Further details of RCA participants and interventional period are described in Table 2.

**Table 2:** Study characteristics of included studies.

First author (year)	Country of study	Setting	Unit	Type of study	Study design	RCA participants	intervention period
Wilson et al.^29^	USA	Mackenzie Health (MH)	all inpatient units	organized an interprofessional falls quality aim committee	Interventional/RCA	− direct care nurses − nurse educators − physiotherapists − occupational therapists − physicians − clinical managers − quality improvement (QI) specialists − patient partners	1 year
Whatley et al.^30^	USA	Children's Hospital at Dartmouth‐Hitchcock Medical Center	mother and baby unit	convened an interprofessional team	Interventional/RCA	− mother‐baby unit − NICU − pediatric unit − adolescent unit	3 years
Ocker et al.^23^	USA	an acute care hospital	adult inpatient behavioral health unit	established a unit‐based QI team	Interventional/RCA	− the behavioral health unit clinical manager − the lead clinical nurse − the executive director of behavioral health services − the chair of the organization's fall prevention collaborative committee − the manager of accreditation and patient safety − the coordinators of accreditation and patient safety	Six months
Kuwaiti & Subbarayalu^31^	Saudi Arabia	King Fahd Hospital of the University	All inpatient units except outpatients, emergency room, obstetric, pediatric, all procedural areas, operating room, and perioperative services	formed a multidisciplinary team	Interventional/RCA	− representatives from the risk management, nursing, physician, operations management, finance and rehabilitation units	1 year
Alexander et al.^32^	USA	Hartford Hospital a Level I Trauma Center	emergency department	revise the existing fall risk tool	Interventional/RCA	− The ED nurse educator − nurse manager − geriatric nurse practitioner	1 year
Weinberg et al.^33^	USA	Staten Island University Hospital	all inpatient units	appointed hospital falls committee (HFC)	Interventional/RCA	− unit managers − staff providing patient care − The HFC co‐chairs	Four‐year
Ruddick et al.^34^	USA	11 hospitals	all inpatient units	conducted the hospitals' interdisciplinary team (IDT)	Interventional/RCA	− representatives from all disciplines that were involved in the patient's care (e.g., nurses, physicians, therapists, and housekeeping) − families and patients	6 months

The root causes discovered for falls involved patient‐related factors (37.5%), environmental factors (25%), organizational and process factors (19.6%), staff and communication factors (17.9%). Table 3 displays the specific categories for the reported root causes. Causes such as medication/sedation, inappropriate footwear, unexpected movement, gait and balance limitations, patients under the influence of alcohol, and cognitive impairments (confusion, disorientation, memory deficits, and dizziness) were identified with higher frequency as contributing patient‐related factors to falls.^23^, ^29^, ^30^, ^32^, ^34^ Various environmental factors have been identified in studies as contributors to falls. These include issues with toilets, tubs, and showers, equipment that poses tripping hazards, a noisy environment, wet floors, cold room temperature, bed/side rails, call lights not being used, crowded rooms, flooring problems, and poor lighting.^23^, ^29^, ^30^, ^34^ The study identified organizational and process factors contributing to falls, including the absence of hourly rounding, improper use of bed alarms, tab alarms, or call bells, an incomplete fall risk assessment tool, and the lack of routine falls risk assessments at admission.^30^, ^31^, ^32^, ^34^ Staff and communication factors can contribute to falls. Some causes related to these factors include a lack of team communication regarding fall risk assessment, inadequate staff accountability to follow fall prevention protocols, and inadequate safety awareness by staff.^23^, ^29^, ^30^, ^31^, ^33^

**Table 3:** Causes of falls occurring in inpatient units.

_Categories_	_Causes_	_Frequency N (%)_
_**Patient‐related factors**_	− _Medication/Sedation,_ ^23^, ^29^, ^30^, ^34^ − _Footwear,_ ^29^, ^34^ − _Unexpected Movement,_ ^29^, ^34^ − _delivery‐related complications and postpartum state_ ^30^ − _hesitancy to accept help_ ^30^ − _Gait, balance limitations,_ ^23^, ^34^ − _Patients tripping on blankets_ ^23^ − _patients under the influence of alcohol,_ ^23^, ^32^ − _bowel/bladder problems_ ^34^ − _changes in clinical condition/electrolyte imbalance_ ^34^ − _confused/disoriented/memory deficits/dizziness,_ ^23^, ^34^ − _hypotension/weakness/fainting_ ^34^ − _seizure_ ^34^	_21 (37.5)_
_**Environmental factors**_	− _Toilet/tub/shower,_ ^29^, ^34^ − _Equipment posed tripping hazards,_ ^29^, ^30^ − _noisy environment,_ ^30^, ^34^ − _wet floors,_ ^23^, ^34^ − _Cold room temperature_ ^23^ − _bed/side rails_ ^34^ − _call light not used_ ^34^ − _crowded room_ ^34^ − _flooring_ ^34^ − _poor lighting_ ^34^	_14 (25)_
_**Organizational and process factors**_	− _Lack of patient visibility from nurses' station_ ^23^ − _Rapid Response Team not called post fall_ ^23^ − _orange sticker/falls risk sign was not used_ ^31^ − _hourly rounding did not happen,_ ^30^, ^31^ − _inappropriate or inadequate use was made of the bed alarms/tab alarms/call bells,_ ^31^, ^32^ − _The incompleteness of the fall risk assessment tool,_ ^31^, ^32^ − _falls risk assessments were not routinely completed at admission,_ ^31^, ^34^	_11 (19.6)_
_**Staff and communication factors**_	− _frequent interruptions prohibited adequate rest_ ^30^ − _Lack of team communication regarding fall risk assessment and interventions,_ ^23^, ^29^ − _details of high fall risk patients were not interchanged between nurses during hand over_ ^31^ − _patient would go to bathroom/toilet without assistance,_ ^31^, ^32^ − _Inadequate staff accountability to follow fall prevention protocols,_ ^30^, ^33^ − _Inadequate safety awareness by staff,_ ^31^, ^33^	_10 (17.9)_

Table 4 outlines the strategies that were reported in included studies to reduce falls. These strategies involved environmental measures and physical protection (29.4%), identifying, and displaying the causes of risk (23.5%), education and culturalization (21.6%), standard fall risk assessment tool (13.7%), and supervision and monitoring (11.8%). No strategies were reported to have resulted in a negative outcome (Appendix A1).

**Table 4:** Strategies used to reduce falls in included studies.

Categories	Strategies	Frequency N (%)
**Environmental measures and physical protection**	− increasing the safety of toileting and mobility^29^ − reduction of potential injury due to a fall by mothers' bed type and bed height^30^ − implement physical preventive interventions including bed rails up, removal of tripping hazards, assuring awake and alert caregivers.^30^ − implementation precautions to minimize injury from falls^30^ − All patients at risk for falls were identified with a yellow fall‐alert bracelet and signs on the door to the patient's room.^23^ − Provided warm sweat suits as an alternative to traditional patient gowns to discourage blanket usage during patient ambulation.^23^ − Wireless bed and chair alarms were purchased for use with impulsive patients.^23^ − bed alarm systems^31^ − orange sticker/fall risk sign in the common fall risk zones in the hospital^31^ − apply a green fall risk bracelet for patient^32^ − Conducted orientation for inpatients and their significant others on the unit's environment and personal equipment, such as call bells, phones, and urinals.^33^ − use of bed and chair alarm^33^ − high‐risk patients be offered assisted toileting every 2 h during the day and at night whenever awake.^33^ − having patients put on their call light when they went to the bathroom^34^ − using informative signs in the patient room^34^	15 (29.4)
**Identifying and displaying the causes of risk**	− Implementation of electronic dashboards to displaying real‐time unit‐specific falls rates^29^ − Implementing a transparent root‐cause analysis process after falls^29^ − increased Patient and family engagement by including them in post‐fall huddles^29^ − Highlighted patients' fall risks on nurses' station whiteboards for easy visibility.^23^ − Incorporated fall risk levels and factors into patient handoff.^23^ − information about current fall risk status to staffs by power chart displaying fall status^31^ − Bulletin boards in the staff break room and in the locker room were used to post a copy of the new tool^32^ − A notice about the reduction of falls was also placed on the ED dashboard.^32^ − When a fall occurred, a post‐fall mini–root cause analysis was conducted with the entire nursing staff^32^ − Nurse managers conducted in‐service meetings emphasizing fall prevention and lessons learned from fall reviews.^33^ − Post fall Assessments by root causes of each fall^33^ − patients and their family members, as well as staff, have contributed useful information toward finding the cause of initial falls that can be used to prevent subsequent falls.^34^	12 (23.5)
**Education and culturalization**	− Facilitating organization‐wide education^29^ − soliciting staff feedback on barriers to falls prevention^29^ − educate parents and staff about the risk of newborn falls^30^ − Implemented unit‐wide staff nurse education module on proper utilization of the Morse Fall Scale (MFS)^23^ − training to staffs^31^ − department‐wide education to introduce a revised ED fall risk assessment^32^ − Creating a culture of fall prevention^32^ − Provided hands‐on and videotaped fall prevention training to resident and attending physicians, rehabilitation medicine therapists, housekeepers, and transporters.^33^ − Daily contests for the lowest number of consecutive fall‐free days were held^33^ − training staff in proper lifting techniques and how to transfer patients^34^ − re‐education of the staff^34^	11 (21.6)
**Standard fall risk assessment tool**	− Employing standardized Morse Fall Risk Assessment tool^29^ − standardize methods of assessing newborn fall risk^30^ − risk factor assessment of falls within 24 h of admission by Morse Fall Scale (MFS)^31^ − incorporated additional measures into their ED fall assessments^32^ − embedded the new fall assessment tool into the triage note^32^ − Fall Risk Assessments On admission^33^ − completion of the initial fall risk assessments and implementation of fall prevention measures^34^	7 (13.7)
**Supervision and monitoring**	− Revised staff workflow to allow the unit staff to maintain a constant presence in the common area.^23^ − monitoring patient medications^29^ − Restriction of Use of Diphenhydramine, Hydroxyzine, and Furosemide^33^ − Rounding protocol^31^ − All at risk patients must have either an exit alarm or a constant observer.^32^ − volunteers (college students) were trained to round on fall risk patients^32^	6 (11.8)

## 4 DISCUSSION

Patient safety involves creating a safe environment and assessing the likelihood of incidents such as falls, medication errors, and infections. On the other hand, RCA is a process that analyzes incidents in a reactive manner to identify contributing factors and propose actions for mitigation.^35^ But like any other method, they are not without limitations. RCA lacks a systemic perspective and implies a single, linear cause. Their inability to see the nonlinear causal mechanism among cause and effect relationships limits them to finding only one absolute root cause.^36^, ^37^ The aim of this study was to identify the causes of falls and effective interventions for their reduction within inpatient healthcare settings through RCA. Indeed, since the included studies were conducted in various treatment facilities and units, it is expected that the causes of falls and the interventions and strategies employed to reduce them would vary.

The studies consistently found that factors related to the patient were the most frequently reported causes of falls. These factors played a significant role in contributing to fall incidents across the various studies. It's important to consider the results of these studies that highlight medication/sedation as a common cause of falls. According to Najafpour et al's case control study, a combination of patient‐related factors and medication history taken into account as risk factors for falls in hospital in‐patients.^38^ In the study by Cioce et al, diuretic drugs were reported as the most common cause of falls with a frequency of 93.33%. To reduce the risk of falls associated with these drugs, recommend rescheduling their administration.^4^ In the study by Santana et al., it was found that 74.8% of fall patients had been prescribed sedatives, psychoactive, or antihypertensive drugs.^39^ According to Ribeiro et al's review, the use of central nervous system drugs increases the risk of inpatient falls, including anxiolytics, hypnotics, sedatives, antipsychotics, opioids, antiepileptics, and antidepressants.^40^ Various medications are linked to a notable risk of falls, known as “fall risk increasing drugs.” Managing drug‐induced falls is crucial for fall prevention, emphasizing the importance of considering the risk level of medications to optimize drug therapy in clinical settings.^41^, ^42^ In the study by Weinberg et al., specific interventions were implemented to address falls related to medication use. One intervention involved restricting the use of diphenhydramine and hydroxyzine in patients aged 65 and above. Another intervention focused on modifying the administration protocol for furosemide. To reduce the need for nighttime voiding, furosemide was administered 3 h earlier, at 6:00 P.M. These interventions were aimed at minimizing the potential side effects and risks associated with these medications, ultimately reducing the likelihood of falls in the targeted patient population.^33^

The studies found that environmental measures and physical protection were the most frequently reported strategies. Addressing these factors is crucial in reducing the risk of falls and promoting a safer environment. In the study conducted by Whatley et al., they implemented measures to reduce the potential injuries caused by falls. These measures included considering the type and height of the mother's bed, implementing physical preventive interventions such as having bed rails up, removing tripping hazards. These interventions aimed to create a safer environment and minimize the risk of falls and subsequent injuries.^30^ Additionally, high‐risk patients were offered assisted toileting every 2 h during the day and whenever awake at night. This intervention aimed to address the increased risk of falls associated with the need for frequent toileting.^33^ In the study conducted by Ocker et al., they implemented various measures to address fall risk among patients. Firstly, they identified all patients at risk for falls by providing them with a yellow fall‐alert bracelet and placing signs on the doors of their rooms. Additionally, they provided warm sweat suits as an alternative to traditional patient gowns, aiming to discourage the excessive use of blankets during patient ambulation, which can increase the risk of falls. Furthermore, wireless bed and chair alarms were purchased and utilized for impulsive patients to provide timely alerts and prevent falls.^23^ In the Kuwaiti and Subbarayalu study, they implemented bed alarm systems and utilized orange stickers/fall risk signs in common fall risk zones within the hospital. These measures were implemented with the goal of reducing falls among patients. Bed alarm systems are designed to alert healthcare providers when a patient attempts to leave the bed without assistance, helping to prevent falls. The use of orange stickers or fall risk signs in specific areas serves as a visual reminder for both patients and staff to exercise caution and take necessary precautions in those areas. By implementing these strategies, the study aimed to enhance patient safety and minimize the incidence of falls in the hospital setting.^31^ In the study by Boot et al., they developed an assessment tool to identify high‐risk patients, used wristbands to identify those at risk of falling, and emphasized increased observation for patients prone to falls.^43^

Identifying and displaying the causes of risk were the second frequently reported strategies. patients and their family members, as well as staff, represent useful information toward finding the cause of initial falls across RCA process that can be used to prevent subsequent falls.^29^, ^32^, ^33^, ^34^ In this regard, various intervention initiatives were implemented, including placing notices on unit dashboards to highlight the reduction of falls,^32^ highlighting patients' fall risks on nurses' station whiteboards for easy visibility, incorporating fall risk levels and factors into patient handoff,^23^ holding daily contests for the lowest number of consecutive fall‐free days,^33^ implementing electronic dashboards to display real‐time unit‐specific falls rates, and soliciting staff feedback on barriers to falls prevention.^29^ Ineffective communication among healthcare team members can result in important information about a patient's fall risk assessment not being properly shared, leading to a lack of awareness and failure to implement preventive measures. When staff members do not feel accountable or responsible for adhering to fall prevention protocols, there is a higher likelihood of gaps in implementing preventive measures, increasing the risk of falls.

Additionally, a lack of sufficient training or education on fall prevention strategies and maintaining a safe environment can contribute to inadequate safety. By prioritizing education, culturalization, and clarity, healthcare organizations can enhance staff knowledge, promote cultural sensitivity, and improve communication practices. These efforts contribute to a safer environment, reduced falls, and improved patient safety outcomes. staff training,^23^, ^29^, ^30^, ^31^, ^32^, ^33^, ^34^ In the study by Paulino et al., it was found that aspects related to the health work process, such as teamwork, professional skills, and nursing care execution, are the main contributing factors to falls. Effective teamwork, proper skills and training, and adherence to protocols are crucial in preventing falls and improving patient safety.^44^ In a review study conducted by Heng et al., it was found that hospital falls prevention interventions that include patient education can effectively reduce falls and associated injuries like bruising, lacerations, or fractures. The success of these interventions is influenced by factors such as the design, delivery method, and quality of educational programs. Well‐designed education programs have shown to improve patients' knowledge and self‐perception of risk, empowering them to take proactive measures to reduce their risk of falling while in the hospital.^45^ One study found that several key factors were identified as helpful in promoting effective patient education. These included providing individualized and consistent education, using small interactive groups, and adopting a patient‐centered multi‐factorial approach.^46^ The findings of a systematic review and meta‐analysis demonstrated that educating patients and staff significantly reduces the occurrence of bed falls in the hospital setting. This highlights the effectiveness of training interventions in preventing such incidents and promoting patient safety.^47^ In addition, according to clinical practice guidelines patients and residents should be screened for fall risks upon admission to the hospital. Multifactorial interventions based on individual risk factors are strongly recommended in hospitals, as they significantly reduce falls. Patients at risk of falling should receive training and advice on fall prevention measures. Caregivers should also receive active educational interventions to increase their knowledge and prevent residents from falling.^48^

## 5 CONCLUSION

Multiple factors, such as patient‐related, environmental, organizational and process, staff and communication factors, contribute to the occurrence of falls in hospitalized patients. Identifying and addressing these factors is essential for preventing falls and ensuring patient safety in healthcare settings. Each healthcare organization has its own unique characteristics and processes, so while certain solutions and strategies have been effective in reducing patient falls in one facility, they may not work the same way in another organization. It is important to consider the specific context and adapt interventions accordingly to ensure effectiveness in different healthcare settings.

## 6 LIMITATIONS

This study has some limitations. As mentioned, due to the limitations of the RCA method, including only studies that used the RCA was one of the major limitations of this study. Secondly, it is difficult to determine which actions were responsible for the reduction in falls, as multiple interventions were implemented simultaneously. finally, detailed information about the characteristics of the population studied could not be obtained. These limitations should be considered when interpreting the study's findings.

## AUTHOR CONTRIBUTIONS

Parvin Lakbala designed the study and its overall methodology, contributed in data analysis and edited the article. Najmeh Bordbar contributed in data gathering, data analysis and prepared the initial draft of the article and also edited the article. Yadolah Fakhri contributed in the methodology of the study and edited the article. All authors read and approved the final manuscript.

## CONFLICT OF INTEREST STATEMENT

The authors declare no conflict of interest.

## TRANSPARENCY STATEMENT

The lead author Najmeh Bordbar affirms that this manuscript is an honest, accurate, and transparent account of the study being reported; that no important aspects of the study have been omitted; and that any discrepancies from the study as planned (and, if relevant, registered) have been explained.

## Data Availability

The data that support the findings of this study are available from the corresponding author upon reasonable request.

## APPENDIX A

Table A1

**Table A1:** Causes of falls and strategies used to reduce falls in included studies.

First author (year)	Fall causes	Falls reducing strategies	Reported fall reduction
Wilson et al.^29^	− Toileting − Patient Related − Medication/Sedation − Footwear − Unexpected Movement − Communication − Staff‐Related − Environment‐Related − ‐ Equipment or Supply	− Facilitating organization‐wide education − Employing standardized Morse Fall Risk Assessment tool − Implementation of electronic dashboards to displaying real‐time unit‐specific falls rates − Implementing a transparent root‐cause analysis process after falls − increasing the safety of toileting and mobility − monitoring patient medications − soliciting staff feedback on barriers to falls prevention − increased Patient and family engagement by including them in post‐fall huddles	from 2.03 to 1.12 per 1000 patient‐days
Whatley et al.^30^	− Mothers' fatigue due to medication − delivery‐related complications and postpartum state − hesitancy to accept help − being accustomed to co‐sleeping − Equipment posed tripping hazards − noisy environment − frequent interruptions prohibited adequate rest − A hospital culture encouraging rooming‐in coupled − lack of unit capability to provide continuous in‐person surveillance − Limited staff's ability to monitor for and address fall risks.	− reduction of potential injury due to a fall by mothers' bed type and bed height − implement physical preventive interventions including bed rails up, removal of tripping hazards, assuring awake and alert caregivers. − implementation precautions to minimize injury from falls − standardize methods of assessing newborn fall risk − educate parents and staff about the risk of newborn falls	from 71.8 to 15.5 per 10,000 births
Ocker et al.^23^	− Inconsistency in fall risk assessment and high‐risk fall patients − Wet floor − Patients tripping on blankets − Cold room temperature − Lack of patient visibility from nurses' station − Lack of team communication regarding patient fall risk and interventions − Rapid Response Team not called post fall − Medications − Gait, balance limitations − Confusion memory deficits − Incontinence	− Revised staff workflow to allow the unit staff to maintain a constant presence in the common area. − Highlighted patients' fall risks on nurses' station whiteboards for easy visibility. − Incorporated fall risk levels and factors into patient handoff. − All patients at risk for falls were identified with a yellow fall‐alert bracelet and signs on the door to the patient's room. − Provided warm sweat suits as an alternative to traditional patient gowns to discourage blanket usage during patient ambulation. − Wireless bed and chair alarms were purchased for use with impulsive patients. − Implemented unit‐wide staff nurse education module on proper utilization of the Morse Fall Scale (MFS)	from 5.43 to 0 per 1000 patient‐days
Kuwaiti & Subbarayalu^31^	− details of high fall risk patients were not interchanged between nurses during hand over − orange sticker/falls risk sign was not used − staff were unaware of what to do for those who were prone to falls − power chart did not display high risk for falls in detail − fall risk assessment was either not completed or omitted at the time of admission − hourly rounding did not happen every 2 h − inappropriate or inadequate use was made of the bed alarms/tab alarms/call bells − patient would go to bathroom without assistance	− Rounding protocol − risk factor assessment of falls within 24 h of admission by Morse Fall Scale (MFS) − bed alarm systems − orange sticker/fall risk sign in the common fall risk zones in the hospital − training to staffs − information about current fall risk status to staffs by power chart displaying fall status	From 6.57 to 1.91 per 1000 patient‐days
Alexander et al.^32^	− need for toileting assistance − patients under the influence of alcohol − Rarely bed or chair exit alarms set − The incompleteness of the fall risk assessment tool	− incorporated additional measures into their ED fall assessments − embedded the new fall assessment tool into the triage note − department‐wide education to introduce a revised ED fall risk assessment − Bulletin boards in the staff break room and in the locker room were used to post a copy of the new tool − A notice about the reduction of falls was also placed on the ED dashboard. − apply a green fall risk bracelet for patient − Creating a culture of fall prevention − When a fall occurred, a post‐fall mini–root cause analysis was conducted with the entire nursing staff − All at risk patients must have either an exit alarm or a constant observer. − volunteers (college students) were trained to round on fall risk patients	Not mentioned
Weinberg et al.^33^	− Inadequate staff accountability to follow fall prevention protocols − Insufficient critical thinking by staff when applying fall prevention protocols − Inadequate safety awareness by staff (fall prevention was a low priority.)	− Provided hands‐on and videotaped fall prevention training to resident and attending physicians, rehabilitation medicine therapists, housekeepers, and transporters. − Conducted orientation for inpatients and their significant others on the unit's environment and personal equipment, such as call bells, phones, and urinals. − Daily contests for the lowest number of consecutive fall‐free days were held − Nurse managers conducted in‐service meetings emphasizing fall prevention and lessons learned from fall reviews. − use of bed and chair alarm − high‐risk patients be offered assisted toileting every 2 h during the day and at night whenever awake. − Restriction of Use of Diphenhydramine, Hydroxyzine, and Furosemide − Fall Risk Assessments On admission − Post fall Assessments by root causes of each fall	From 3.9 to 1.1 per 1000 patient‐days
Ruddick et al.^34^	− staff were not routinely completing falls risk assessments at admission, − staff were not were they completing the post‐fall assessment tools. − bed/side rails − call light not used − crowded room − foot wear − flooring − tub/shower − wet floors − noise level − poor lighting − bowel/bladder problems − changes in clinical condition − confused/disoriented − dizziness − hypotension − intentional act − loss of balance − seizure − medications − electrolyte imbalance − weakness/fainting	− having patients put on their call light when they went to the bathroom − using informative signs in the patient room − training staff in proper lifting techniques and how to transfer patients − patients and their family members, as well as staff, have contributed useful information toward finding the cause of initial falls that can be used to prevent subsequent falls. − re‐education of the staff − completion of the initial fall risk assessments and implementation of fall prevention measures	from 133.9 to 73.05 per 1000 patient‐days
